# The effect of two multi-component behavior change interventions on cognitive functions

**DOI:** 10.1186/s12889-022-13490-5

**Published:** 2022-05-31

**Authors:** Emil Bojsen-Møller, Rui Wang, Jonna Nilsson, Emerald G. Heiland, Carl-Johan Boraxbekk, Lena V. Kallings, Maria Ekblom

**Affiliations:** 1 Department of Physical Activity and Health, The Swedish School of Sport and Health Sciences, GIH, Stockholm, Sweden; 2 Division of Clinical Geriatrics, Department of Neurobiology, Care Sciences and Society, Karolinska Institutet, Stockholm, Sweden; 3 Department of Surgical Sciences, Medical Epidemiology, University of Uppsala, Uppsala, Sweden; 4 Danish Research Centre for Magnetic Resonance (DRCMR), Centre for Functional and Diagnostic Imaging and Research, Copenhagen University Hospital - Amager and Hvidovre, Copenhagen, Denmark; 5 Department of Radiation Sciences, Diagnostic Radiology, Umeå University, Umeå, Sweden; 6 Institute of Sports Medicine Copenhagen (ISMC) and Department of Neurology, Copenhagen University Hospital Bispebjerg, Copenhagen, Denmark; 7 Institute for Clinical Medicine, Faculty of Medical and Health Sciences, University of Copenhagen, Copenhagen, Denmark; 8 Department of Neuroscience, Karolinska Institutet, Stockholm, Sweden

**Keywords:** Physical activity, Sedentary behavior, Office workers, Cognitive function, Workplace, Intervention

## Abstract

**Background:**

We previously reported the effects of two cluster-randomized 6-month multi-component workplace interventions, targeting reducing sedentary behavior or increasing physical activity among office workers, on movement behaviors and cardiorespiratory fitness. The primary aim of this study was to investigate the effects of these interventions on cognitive functions compared to a wait-list control group. The secondary aims were to examine if changes in cognition were related to change in cardiorespiratory fitness or movement behaviors and if age, sex, or cardiorespiratory fitness moderated these associations.

**Methods:**

Both interventions encompassed multi-components acting on the individual, environmental, and organizational levels and aimed to change physical activity patterns to improve mental health and cognitive function. Out of 263 included participants, 139 (mean age 43 years, 76% females) completed a neuropsychological test battery and wore accelerometers at baseline and 6-month follow-up. The intervention effect (aim 1) on cognitive composite scores (i.e., Executive Functions, Episodic Memory, Processing Speed, and Global Cognition) was investigated. Additionally, associations between changes in movement behaviors and cardiorespiratory fitness, and changes in cognition were examined (aim 2). Moreover, age, sex, and cardiorespiratory fitness level were investigated as possible moderators of change associations (aim 3).

**Results:**

Overall, cognitive performance improved from baseline to follow-up, but the change did not differ between the intervention groups and the control group. Changes in cardiorespiratory fitness or any movement behavior category did not predict changes in cognitive functions. The association between changes in time in bed and changes in both Executive Function and Global Cognition were moderated by age, such that a more positive relation was seen with increasing age. A less positive association was seen between changes in sedentary behavior and Processing Speed for men vs. women, whereas higher cardiorespiratory fitness was related to a more positive association between changes in moderate-intensity physical activity and Global Cognition.

**Conclusion:**

The lack of an intervention effect on cognitive functions was expected since the intervention did not change movement behavior or fitness. Age, sex, and cardiorespiratory fitness level might moderate the relationships between movement behaviors and cognitive functions changes.

**Trial registration:**

ISRCTN92968402. Registered 09/04/2018.

## Introduction

Office workers are at risk of accumulating high amounts of sedentary behavior during work and leisure time [1, 2]. Sedentary behavior (SED), defined as any waking behavior with an energy expenditure of less than 1.5 metabolic equivalents while in a sitting or reclining posture, is related to increased risk of cardiovascular disease [3], increased risk of dementia [4], and worse cognitive function [5].

Multiple attempts have been made to identify strategies to reduce SED, increase moderate-to-vigorous physical activity (MVPA), or combine both among office workers [6–9]. Still, most studies used single-component interventions, and the quality of evidence favoring such interventions is low [9]. Behavior change interventions have been suggested to be more effective when containing multiple components acting on several levels (e.g., organizational, environmental, and individual) than interventions working on only one level [10].

Structured physical activity interventions show positive effects on general cognitive function, although small, but also in specific domains [11]. While most evidence stems from investigations in older adults [12], research in a working-age population is limited. This lack of knowledge hinders employers and occupational health promoters from providing evidence-based support for office workers. A recent study targeting overweight and obese office workers reported that installing treadmill workstations did not significantly affect cognitive functions, but increased walking time was associated with increased hippocampus volume at 13 months follow-up [13]. Increased hippocampus volume has previously been associated with improved cognitive function, accompanied by increased cardiorespiratory fitness in older adults [14]. Instead of installing treadmills, reducing prolonged SED may be a more feasible and sustainable way to improve health outcomes in the workplace [15]. Frequent bouts of light-intensity physical activity improve glycemic control [16], reduce blood pressure [17], eases fatigue [18], and improves cognitive functioning [19]. In addition, frequent, short breaks of moderate-intensity walking may improve mood and alertness compared with sitting uninterrupted for 3-hours [20]. However, workplace interventions aiming to decrease SED or increase physical activity often fail to facilitate behavior change [6, 9]. Thus, from the perspective of employers, it is still unclear how we should design multi-component workplace interventions to improve movement behavior in a way that supports healthy brain functions, i.e., mental health and cognitive function.

We designed [21] and recently published the results of a multi-component three-armed cluster-randomized ecological trial, investigating the effectiveness of two interventions to reduce SED or increase MVPA to improve mental health and cognitive function in healthy office workers [22, 23]. The results revealed that the interventions were unsuccessful in changing device-measured movement behavior. Neither the intervention directed towards increasing physical activity (iPA) nor that directed towards reducing SED (iSED) resulted in any behavior change at any intensity compared to the control group [22, 23]. However, it is still unknown if the interventions influenced our distal outcomes, such as cognitive function and mental health [21].

Therefore, the first aim of this study was to investigate the effect of two multi-component interventions aiming at increasing MVPA or reducing SED on cognitive function. The second aim was to examine if changes in any movement behavior category or cardiorespiratory fitness were related to changes in cognitive function. The third aim was to explore if age, sex, or cardiorespiratory fitness moderated the associations between changes in movement behavior and changes in cognitive function.

## Methods

### Participants

**Fig. 1:**
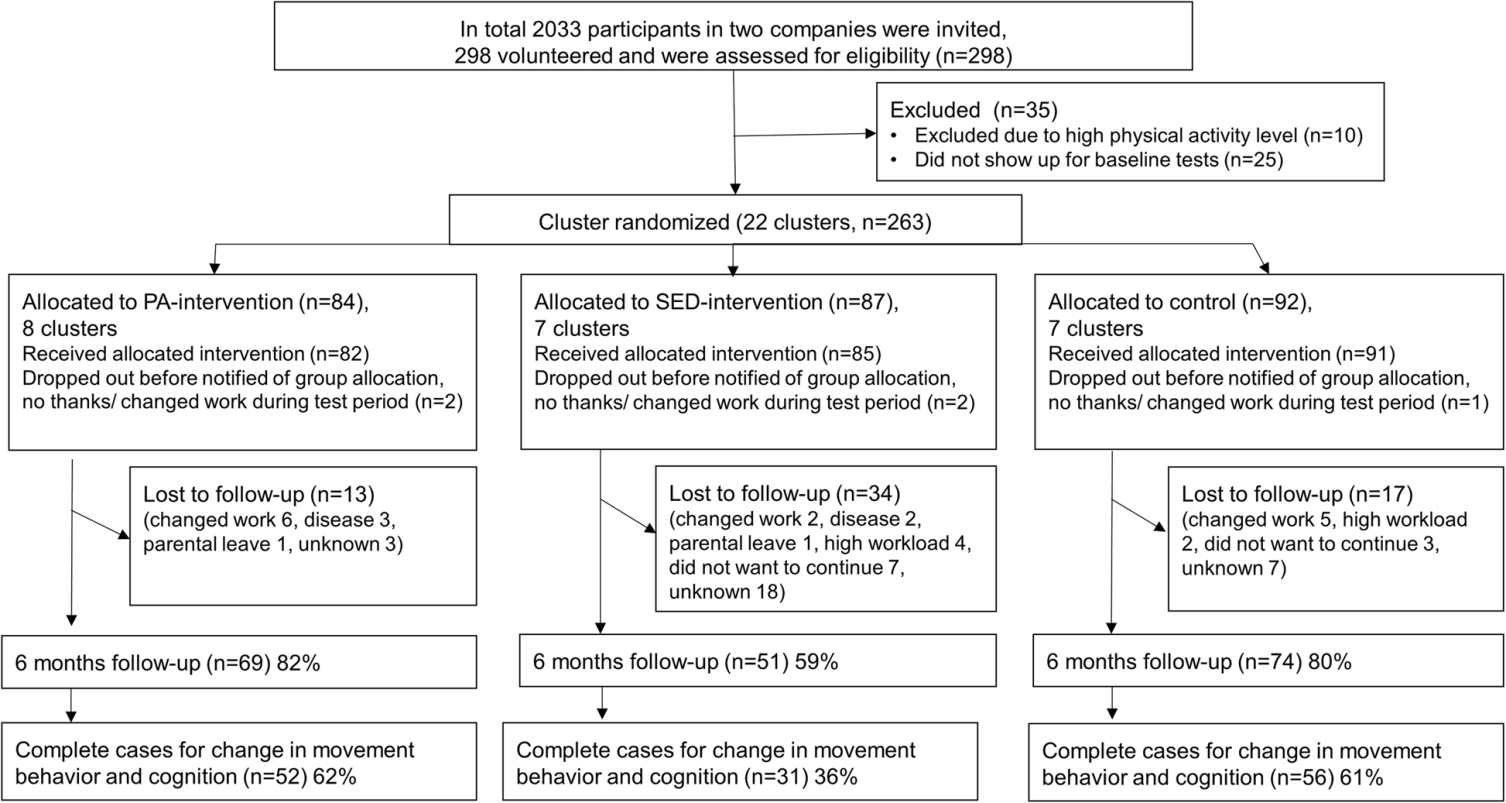
Flow chart of participant enrollment

Two-thousand-thirty-three office workers at two Swedish companies were invited by email. After receiving written and verbal information about the study, participants signed informed written consent and were then included (see [23] for more details on the recruitment procedure). Out of those invited, 298 were screened for participation. Individuals that exceeded 30 min/day of MVPA in prolonged bouts (≥10 min), measured with accelerometers, were excluded (*n* = 35). The remaining 263 individuals were assigned to a cluster, and each cluster was then randomized into one of three groups. Clusters were created based on participants that had the same line manager, had group meetings frequently and limited meetings with other work groups. In total, 139 participants completed cognitive, activity monitoring, and fitness testing at baseline and 6-month follow-up. Employers permitted participants to participate in the study during working hours. See Fig. 1 for a flow chart of the enrollment, participation, and analysis of the sample.

### Study design

The study was a 6-month, three-armed cluster randomized controlled trial in which participants were randomly assigned to an intervention aiming to increase MVPA (iPA), an intervention aiming to reduce SED (iSED), or a wait-listed control (CON). A detailed description of the study design, intervention, and randomization has been published elsewhere [21]. In short, both interventions were based on the ecological framework for health behavior change, addressing multiple levels, such as the individual, environmental, and organizational levels [10]. Both interventions consisted of motivational counseling at the individual level using Cognitive Behavioral Therapy (CBT) and Motivational Interviewing (MI) in combination. The essential components in CBT treatment consist of challenging negative feelings and thoughts to change the behavior. Through active collaboration with a counselor, the individual identifies barriers and facilitators to create a realistic picture of the demands of the behavior change [24]. MI is an individual-centered counseling technique targeting facilitating behavior change [25]. The aim of counseling differed between the interventions. The counseling of iPA aimed to increase MVPA, while the counseling of iSED aimed to reduce SED and break up prolonged periods of sitting. At the environmental level, participants in the iPA had access to a commercial gym for 6-months, whereas the iSED group had organized standing and walking meetings. Each cluster had a team leader responsible for organizing walking and standing meetings in the iSED group and exercise sessions and lunch walks in the iPA group. At the organizational level, team leaders encouraged employees to increase MVPA or reduce SED for the iPA and the iSED, respectively. At baseline and after 6-months, participants completed neuropsychological testing, accelerometry-based measurement of movement behaviors, and cardiorespiratory fitness testing.

### Neuropsychological test battery

The neuropsychological test battery was a modified version of the battery used in Jonasson et al. (2017) [26]. The battery consisted of seven computerized and four non-computerized tests, and it took approximately 1 hour to complete the battery. The test order was: Trail Making Test A, Trail Making Test B, Automated operation span, Recognition: encoding, Digit Symbol, n-back, Free Recall, Digit Span Backward, Recognition: recall, and Stroop color and word test. At follow-up, Free Recall and Word Recognition words changed to reduce learning effects.

#### Trail-making task a (TMT-A)

Participants connected circles containing numbers 1 to 25 as fast as possible using a pen. The test leader corrected the first mistake only. The outcome variable was the time it took to complete the test in seconds.

#### Trail-making task B (TMT-B)

Participants were instructed to connect circles with numbers and letters written on a piece of paper, alternating between numbers and letters, in the proper order, as fast as possible. E.g., 1-A-2-B. The test leader corrected the first mistake only. The outcome variable was the time it took in seconds to connect all circles correctly.

#### Automated operation span

The objective of the test was to remember letter sequences as correctly as possible. In between each letter, participants solved a simple mathematical task. At the end of each set, participants placed the letters in the correct order using the computer mouse. The letter sequence length varied from 3 to 7 letters. The outcome variable was the number of perfectly remembered sets [27].

#### Word recognition

Thirty words were shown for 3 seconds, each interspaced by 1 second. After approximately 25 minutes, a delayed retention test was performed. Participants were asked to indicate with the keypress whether they had seen the word before. The outcome variable was how many words were correctly recognized [28].

#### Digit symbol

The test’s objective was to identify a specific digit-symbol combination on a screen. A row of digit-symbol combinations was displayed on the top of the screen, with a single reference digit/symbol combination underneath. Participants identified with the arrow keys if the reference combination appeared or not in the row of combinations. The outcome variable was the average reaction time in milliseconds on correct responses [29].

#### N-back

On a screen, one number in a sequence was shown. Within 2 seconds from the onset, participants identified if the number was the same as they saw one number before (1-back) or three numbers prior (3-back). The outcome variables were the average reaction time in milliseconds on correct trials for 1-back and the number of correct trials (accuracy) for 3-back [30].

#### Free recall

Sixteen Swedish nouns were visually presented on a screen for 2 seconds, each with an interval of 1 second. Immediately after, participants were asked to write down as many words as they recalled. The outcome variable was the number of words recalled correctly [31].

#### Backward digit span

One digit at the time was shown in a sequence on the screen for 1 second with an interval of 250 ms. The task was to write the numbers in the backward order using the number keys on the keyboard. The sequence length started with two digits and continued until participants made two mistakes on the same sequence length. The outcome variable was the sequence length of the longest completed sequence.

#### Stroop

On a piece of paper, 50 words of four different colors were written. The task was to say the printed colors of the words and not read the written colors. The outcome variable was the time it took in seconds to correctly state the colors of the written words [32].

### Movement behaviors

Participants wore an ActiGraph™ GT3X tri-axial accelerometer for seven consecutive days at baseline and the 6-month follow-up. The accelerometer was placed on the right hip during the daytime and moved to the right wrist during bedtime. Participants were not allowed to wear the accelerometer during water activities. Participants filled in an activity- and sleep diary to obtain information on wake time, time in bed, work time, and leisure time. A standard wake time from 6 AM to 11 PM was assumed when diary information was missing. We processed accelerometer data using MATLAB R2020a (MathWorks, Natick, MA, USA). We analyzed time spent in SED, light (LPA), moderate (MPA), and vigorous physical activity (VPA). Intensity metrics from the three axes were based on non-filtered raw accelerations with a 10 Hz low-pass filter [33] and a three-second epoch length united into vector magnitude. Zero output for at least 60 seconds was defined as non-wear time with allowance for maximal 2 min above zero but below the sedentary cut-point [34]. We classified intensity levels into different energy expenditure categories based on metabolic equivalents (METs) ranges, i.e., SED (< 1.5 METs), LPA (≥1.5 to < 3 METs), MPA (≥3 to < 6 METs), and VPA (≥6 METs) [33]. Time spent in bed, SED, LPA, MPA, and VPA were linearly scaled to 1440 min using the compositional package [35] in R studio (version 4.0.2, 36]. Then, we calculated compositional means of time spent in these five behaviors. Each participant’s composition was then transformed into five sets of four isometric log-ratios (See [22] for more details). The 1st isometric log-ratio (ilr) of each set (describing one movement behavior in relation to the others) was then used as a compositional measure of the movement behaviors in all subsequent analyses and are from now on referred to as ilr_1_ Time in bed, ilr_1_ SED, ilr_1_ LPA, ilr_1_ MPA, and ilr_1_ VPA.

### Cardiorespiratory fitness

We used a submaximal cycle ergometer test to estimate both relative (mL·kg^− 1^·min^− 1^) and absolute (L/min) maximal oxygen uptake (VO_2max_). With a pedaling rate of 60 rpm, participants biked for 4 min at a standardized low intensity of 0.5 kilopounds. Subsequently, participants biked on an individualized higher work rate for an additional 4 min. To estimate VO_2max_ we applied the heart rate difference between intensities, divided by the work rate differences, to the equation [36].

### Demographic variables

Participants filled out a questionnaire containing demographic information (e.g., age, sex, and years of education), and length and weight were measured at baseline.

### Data processing

#### Cognitive outcomes

First, we unified all neuropsychological test scores so that ascending scores related to better performance. Then, standardized z-scores were calculated for each cognitive test score at each time point using mean and standard deviation from baseline for the respective test. We defined four latent constructs. The Executive Functions (EF) construct was comprised of Stroop, TMT-B, 3-back, backward digit span, and automated operation span. Processing Speed (PS) comprised TMT-A, 1-back (reaction time), and Digit Symbol. Episodic Memory (EM) included Free Recall and Word Recognition; and a Global Cognition score comprising all tests combined. Using the Lavaan package in R, we first tried to fit a latent change score model for each identified latent cognitive construct as suggested by Kievit et al. (2018). Because of the problem of Heywood cases in our models, we applied the conventional cognitive composition scores of t-scores as outcomes. T-scores were calculated by multiplying each tests’ z-scores with 10 and adding 50. The sum of all tests divided by the number of tests constitutes each cognitive domains’ composition score.

### Statistical analysis

All statistical analyses were conducted in R studio (version 4.0.2) [37]. We carried out analyses in all complete cases of 139 participants who performed the neuropsychological test battery at baseline and at the 6-month follow-up. To understand the selection bias, participants who dropped out were compared to participants who completed the intervention at baseline, using unpaired Student’s t-tests and Chi-square tests for sex distribution. Same statistical methods were employed for comparing the intervention groups and the control group.

To examine the intervention effects (aim 1), linear mixed models were performed using z-composite scores of the cognitive domains as the outcome variables. In the linear mixed models, we estimated the fixed effects of intervention groups, time, and the interaction between group and time, after considering the random effect of intercept, slope, as well as clusters. In addition, all models included age, sex, and education as covariates. Within-group changes were analyzed using Tukey pairwise comparisons.

By adding a two-way interaction between changes in behavior (i.e., fitness or movement behavior ilrs) and time to the linear mixed model used for aim 1, associations between changes in movement behaviors and cardiorespiratory fitness and changes in each cognitive domain (aim 2) were investigated, after adjusting for baseline level of behaviors and the change of behaviors from baseline.

Examination of how age, sex, or fitness level moderated the relationships between changes in cognition and changes in movement behavior (aim 3) was done by checking the three-way interactions between time, change in movement behavior, and the moderators (i.e., age, sex, or fitness) in the linear mixed models. For both aim 2 and aim 3 the three groups were collapsed.

### Sensitivity analysis

Participants who had participated in a previous study containing similar neuropsychological tests were compared to participants who completed the battery for the first time. The comparison was made at baseline and at the 6-month follow-up, using unpaired Student’s t-tests.

## Results

Table 1 displays the subject characteristics. Analysis of the subject characteristics showed that the control group was less fit than the iSED group but did not differ from the iPA group. Participants who dropped out of the study were younger, performed poorer on EM, and were more likely to be in the iSED group than compliant subjects. Five participants missed data on EF, one participant on EM, one participant on PS, and 6 participants on Global Cognition.

**Table 1:** Baseline subject characteristics

	Control	iPA	iSED
Age, mean years (SD)	45 (7)	41 (9)	42 (8)
Sex, % male	23	23	29
Education, mean years (SD)	15 (2)	15 (2)	15 (2)
Body mass index, kg/m2, mean (SD)	27 (5)	25 (4)	24 (4)
Time in bed, minutes (%)	464 (33)	460 (32)	461 (32)
Sedentary, minutes (%)	763 (53)	764 (53)	767 (53)
Light physical activity, minutes (%)	107 (7)	109 (8)	107 (7)
Moderate physical activity, minutes (%)	98 (7)	98 (7)	95 (7)
Vigorous physical activity, minutes (%)	9 (1)	10 (1)	11 (1)
VO_2max_	35 (7)	37 (7)	39 (7)^a^
N	52	56	31

*SD* standard deviation, *iPA* intervention group to increase moderate-to-vigorous physical activity, *iSED* intervention group to reduce sedentary behavior

^a^ a significant difference from the control group

### Intervention effects

**Fig. 2:**
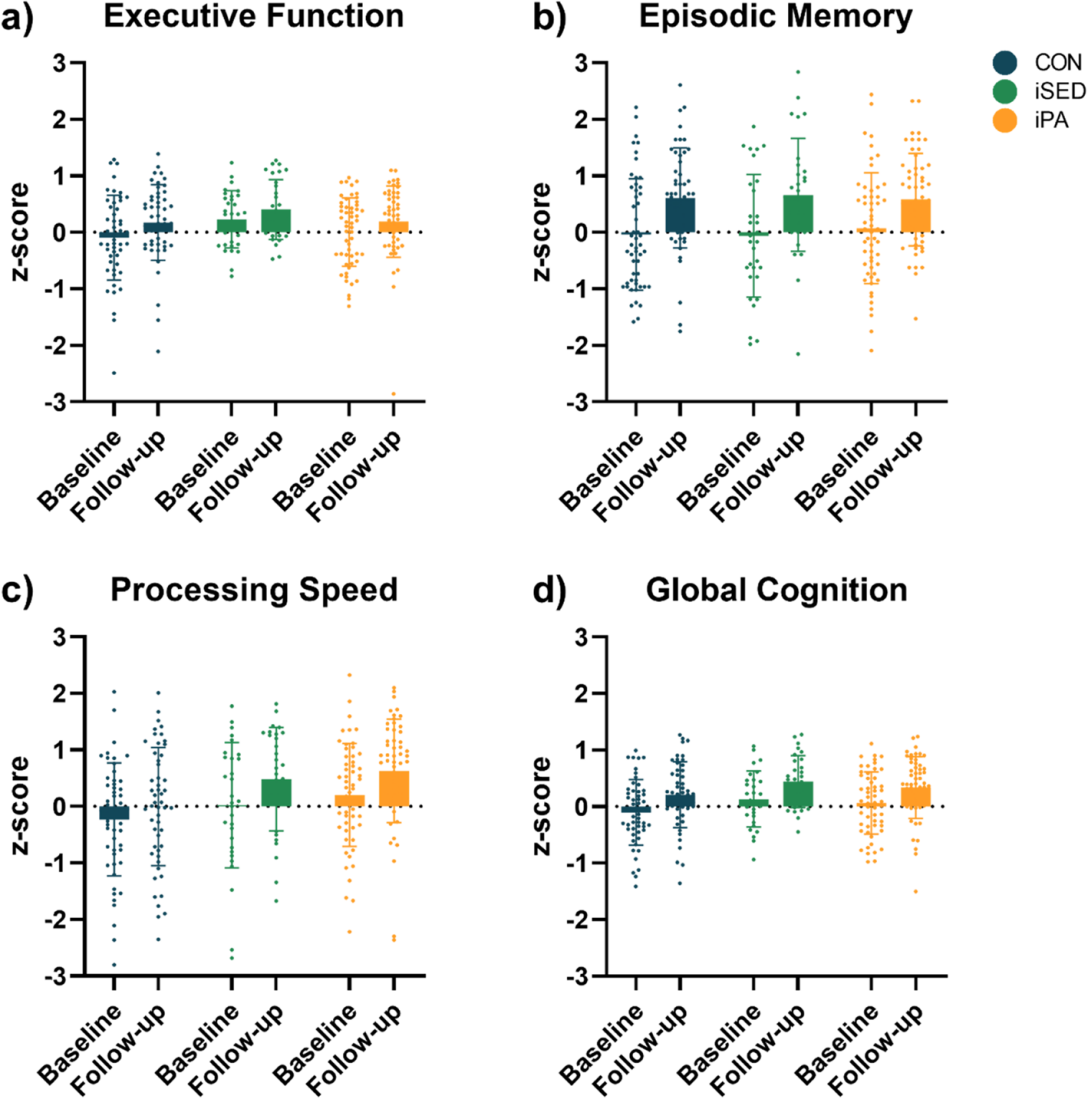
Mean cognitive functions (z-scores with standard deviations) at baseline and follow-up plotted with individual values. **a** Executive Functions (EF) comprised of: Stroop, TMT-B, 3-back, backward digit span, and automated operation span. **b** Episodic Memory (EM) included: Free Recall and Digit symbols. **c** Processing Speed (PS) comprised of: TMT-A and Digit Symbol. **d** Global Cognition combining all tests. iPA = intervention group to increase moderate-to-vigorous physical activity; iSED = intervention group to reduce sedentary behavior; CON = control group

Figure 2 shows the results from the cognitive domains at baseline and follow-up. Analysis of the RCT-effect (aim 1) revealed no effect of the intervention on any of the cognitive domains (see Table 2). All groups improved performance significantly on EM and PS from baseline to follow-up. The iPA and control group improved EF from baseline to follow-up, whereas the iSED did not (see Table 3).

**Table 2:** Differences in change from baseline to 6-month follow-up between groups for the cognitive domains

*Between-group differences*	iPA vs control	iSED vs control	iPA vs iSED
	*Estimate (95%CI)*	*Estimate (95%CI)*	*Estimate (95%CI)*
Executive function	−0.811 (−2.83–1.20)	−0.959 (− 3.33–1.41)	0.148 (− 2.19–2.49)
Episodic memory	− 1.205 (− 4.86–2.45)	0.693 (− 3.59–4.98)	− 1.897 (− 6.14–2.35)
Processing speed	1.506 (−0.40–3.41)	1.819 (− 0.45–4.09)	− 0.313 (− 2.55–1.93)
Global Cognition	−0.349 (− 1.80–1.10)	−0.015 (− 1.74–1.71)	−0.334 (− 2.03–1.36)

Estimates with 95% confidence intervals from the linear mixed models. Models contained age, sex, and education as fixed effects. Cluster and subject were entered as random effects. Cognitive domains consisted of t-composite scores

*iPA* intervention group to increase moderate-to-vigorous physical activity, *iSED* intervention group to reduce sedentary behavior

**Table 3:** Differences in change from baseline to 6-month follow-up within each group for the cognitive domains

*Change within the intervention groups*	iPA	iSED	Control
	*Estimate (95%CI)*	*Estimate (95%CI)*	*Estimate (95%CI)*
Executive function	1.892 (0.47–3.32)^a^	1.743 (− 0.17–3.66)	2.703 (1.22–4.18)^a^
Episodic memory	4.460 (1.86–7.06)^a^	6.358 (2.89–9.82)^a^	5.665 (2.99–8.34)^a^
Processing speed	3.284 (1.93–4.63)^a^	3.597 (1.75–5.44)^a^	1.778 (0.38–3.18)^a^
Global Cognition	2.776 (1.75–3.80)^a^	3.110 (1.71–4.51)^a^	3.125 (2.06–4.19)^a^

Estimates with 95% confidence intervals from the linear mixed models. Models contained age, sex, and education as fixed effects. Models considered random intercept, random slope, and random cluster. Cognitive domains consisted of t-composite scores

*iPA* intervention group to increase moderate-to-vigorous physical activity, *iSED* intervention group to reduce sedentary behavior

^a^Significantly changed from baseline to 6-months follow-up

### Change-change analysis

The change-change analysis (aim 2) showed that changes in fitness or any movement behavior ilr_1_ in the total sample were not related to changes in any of the cognitive domains (see Table 4).

**Table 4:** Associations between changes in cognitive functions and changes in fitness/movement behaviors

	Δ Executive Function	Δ Episodic Memory	Δ Processing Speed	Δ Global Cognition
	*Estimates (95%CI)*	*Estimates (95%CI)*	*Estimates (95%CI)*	*Estimates (95%CI)*
Δ VO_2max_ (mL·kg^− 1^·min^− 1^)	− 0.00 (− 0.12–0.12)	− 0.04 (− 0.24–0.16)	−0.02 (− 0.14–0.10)	− 0.00 (− 0.09–0.08)
Δ VO_2max_ (L/min)	0.28 (−1.40–1.95)	−1.89 (− 4.71–0.92)	− 0.65 (− 2.30–1.00)	−0.24 (− 1.46–0.99)
Δ ilr_1_ Time in bed	−1.17 (− 3.13–0.78)	0.08 (− 3.15–3.31)	0.46 (−1.41–2.34)	−0.79 (− 2.22–0.63)
Δ ilr_1_ Sedentary behavior	−0.53 (− 2.47–1.41)	−2.04 (− 5.34–1.27)	1.70 (−0.20–3.60)	−0.24 (− 1.66–1.17)
Δ ilr_1_ Light physical activity	−0.48 (− 2.29–1.33)	−0.91 (− 3.98–2.16)	−0.10 (− 1.88–1.68)	−0.32 (− 1.63–1.00)
Δ ilr_1_ Moderate physical activity	0.61 (−1.12–2.33)	1.09 (− 1.87–4.05)	−0.31 (− 2.01–1.39)	0.50 (− 0.75–1.76)
Δ ilr_1_ Vigorous physical activity	0.18 (− 0.54–0.89)	0.22 (−0.99–1.43)	−0.23 (− 0.93–0.47)	0.09 (− 0.43–0.61)
No. of observations	134	138	138	133
* p < 0.05				

Linear mixed model estimates of the two-way interactions between changes in ilr_1_ movement behavior (i.e., Time in bed, Sedentary behavior, and Light, Moderate, and Vigorous physical activity) and changes in cognitive domain (i.e., Executive function, Episodic memory, and Processing speed) with 95% confidence interval. Significant moderations are marked with *

### Moderation analysis

Explorative moderation analysis (aim 3) showed that sex, age, and cardiorespiratory fitness significantly moderated some relations between movement behaviors and cognition changes (see Table 5). Age moderated the association between changes in ilr_1_ Time in bed and changes in EF (β = 0.26, 95% CI [0.03 to 0.49]) and Global Cognition (β = 0.26, 95% CI [0.09 to 0.42]). Increasing age was associated with a more positive association between changes in ilr_1_ Time in bed and EF and Global Cognition changes. Age did not moderate any other change on change association. Sex moderated the relationship between changes in ilr_1_ SED and changes in PS. Being male versus female was associated with a less positive relation between ilr_1_ SED and PS changes (β = − 4.69, 95% CI [− 8.80 to − 0.58]). Baseline cardiorespiratory fitness moderated the association between changes in ilr_1_ MPA and changes in Global Cognition. Higher cardiorespiratory fitness was associated with a more positive association between changes in ilr_1_ MPA and changes in Global Cognition (β = 0.19, 95% CI [− 0.00 to − 0.37]).

**Table 5:** Moderation of age, sex, and fitness on the association between cognitive functions and movement behavior changes

	Δ Executive Function	Δ Episodic Memory	Δ Processing Speed	Δ Global Cognition
*Moderation of age*	*Estimates (95%CI)*	*Estimates (95%CI)*	*Estimates (95%CI)*	*Estimates (95%CI)*
Δ ilr_1_ Time in bed	0.26 * (0.03–0.49)	0.19 (− 0.17–0.55)	0.13 (− 0.08–0.34)	0.26 * (0.09–0.42)
Δ ilr_1_ Sedentary behavior	0.08 (− 0.17–0.34)	0.35 (− 0.06–0.77)	0.04 (− 0.20–0.29)	0.13 (− 0.06–0.31)
Δ ilr_1_ Light physical activity	0.09 (− 0.13–0.31)	0.11 (− 0.25–0.46)	0.04 (− 0.17–0.26)	0.09 (− 0.07–0.25)
Δ ilr_1_ Moderate physical activity	−0.24 (− 0.49–0.00)	0.08 (− 0.34–0.50)	− 0.19 (− 0.43–0.05)	− 0.14 (− 0.32–0.04)
Δ ilr_1_ Vigorous physical activity	− 0.03 (− 0.12–0.07)	−0.10 (− 0.26–0.05)	−0.02 (− 0.11–0.07)	−0.05 (− 0.11–0.02)
*Moderation of sex*				
Δ ilr_1_ Time in bed	3.61 (− 0.79–8.02)	−3.97 (− 11.50–3.55)	−3.57 (− 7.94–0.81)	0.24 (− 3.00–3.49)
Δ ilr_1_ Sedentary behavior	3.63 (− 0.56–7.82)	−3.01 (− 10.16–4.15)	− 4.69 * (− 8.80 – − 0.58)	−0.06 (− 3.15–3.04)
Δ ilr_1_ Light physical activity	2.42 (− 1.63–6.47)	− 2.05 (− 8.91–4.80)	− 1.26 (− 5.30–2.78)	0.49 (− 2.47–3.46)
Δ ilr_1_ Moderate physical activity	− 0.90 (− 4.50–2.70)	2.11 (− 4.03–8.25)	1.03 (− 2.57–4.64)	0.51 (− 2.13–3.14)
Δ ilr_1_ Vigorous physical activity	−1.17 (− 2.70–0.37)	0.77 (− 1.85–3.40)	1.11 (− 0.41–2.63)	−0.23 (− 1.36–0.89)
*Moderation of Fitness*				
Δ ilr_1_ Time in bed	−0.04 (− 0.29–0.21)	−0.36 (− 0.76–0.04)	−0.09 (− 0.32–0.15)	−0.14 (− 0.32–0.04)
Δ ilr_1_ Sedentary behavior	0.14 (− 0.10–0.38)	− 0.39 (− 0.79–0.01)	0.08 (− 0.16–0.31)	−0.00 (− 0.18–0.17)
Δ ilr_1_ Light physical activity	0.06 (− 0.14–0.27)	− 0.03 (− 0.37–0.32)	0.00 (− 0.20–0.21)	0.02 (− 0.13–0.17)
Δ ilr_1_ Moderate physical activity	0.20 (−0.06–0.45)	0.06 (− 0.38–0.50)	0.19 (−0.07–0.44)	0.19 * (0.00–0.37)
Δ ilr_1_ Vigorous physical activity	−0.05 (− 0.14–0.03)	0.09 (− 0.06–0.23)	−0.02 (− 0.11–0.07)	−0.01 (− 0.08–0.06)
No. of observations	134	138	138	
* p < 0.05				

Linear mixed model estimates of the three-way interactions between changes in movement behavior ilr_1_ (i.e., Time in bed, Sedentary behavior, and Light, Moderate, and Vigorous physical activity), changes in cognitive domain (i.e., Executive function, Episodic memory, and Processing speed) and the moderator (i.e., age, sex, or fitness) with 95% confidence interval. Significant moderations are marked with *

### Sensitivity analysis

Participants that previously had participated in a study containing similar cognitive tests did not perform significantly differently from the rest of the sample at baseline or follow-up. Moreover, cognitive changes from baseline to follow-up did not differ in this sub-sample.

## Discussion

In the present study, we investigated how two 6-month multi-component cluster-randomized interventions aimed at changing MVPA or SED influenced cognitive function in office workers. The results showed that the two interventions were unsuccessful in changing cognitive functions compared to the control group. Moreover, changes in cardiorespiratory fitness or any movement behavior ilr_1_ did not relate to changes in cognitive functions. Explorative moderation analyses showed that age moderated the relationship between changes in ilr_1_ Time in bed and changes in EF. Moreover, the relationship between changes in ilr_1_ SED and PS were moderated by sex, whereas cardiorespiratory fitness moderated the associations between changes in ilr_1_ MPA and Global Cognition.

In a recent meta-regression of 80 randomized controlled trials, structured exercise interventions showed a small but positive effect on multiple cognitive domains [11]. However, such structured exercise may not be feasible and sustainable in a real-life setting. Behavior change research suggests that persons motivated by their own needs and desires are more likely to sustain a new healthy behavior [36], and structured exercise interventions may not fulfill this. Therefore, it is crucial to investigate the effect of sustainable behavior change interventions supporting the transition towards and maintenance of the new behavior. The present trial is the first to investigate how two multi-component, ecological, cluster-randomized workplace interventions aiming at different movement behaviors influence cognitive functions.

We recently showed that the two interventions were ineffective in changing cardiorespiratory fitness or any movement behavior during work or leisure time [22]. Here we present the effect of the intervention on cognitive functions. We found that the interventions were ineffective in changing cognitive functions. The interventions were designed to explicitly target increasing MVPA or reducing SED to improve office workers’ mental health and cognitive functions [21]. While our findings suggest that the investigated 6-month multi-component interventions did not affect cognitive functions, the lack of intervention effect on physical activity and SED impaired our ability to evaluate how successful changes in physical activity and sedentary behavior causally affect cognitive functions.

Participants’ physical activity level when entering the study may explain the lack of effect on cognitive functions. Unfortunately, the recruitment of too active participants may have limited the potential effect of the intervention [22], making it difficult for participants to increase physical activity further and thus enhance cognitive functions. We intended to recruit only inactive participants [21]. Therefore, we excluded persons with more than 30 min/day of MVPA in bouts of 10 min. Despite our attempts, the participants enrolled in the study were physically active [23], which is further supported by good cardiorespiratory fitness levels [22] compared to population data [38]. On average, participants performed 97 (SD ± 23) minutes of MPA/day, compared to a reported 32 minutes of MPA/day in a meta-analysis containing studies of office workers [39]. Notably, in the present study, accelerometer data has been processed using non-filtered raw accelerations with a 10 Hz low-pass filter and a three-second epoch length united into vector magnitude [33], which results in higher levels of MPA. However, even with a more traditional data processing approach, presented in the effectiveness study of the trial, the participants included were highly physically active [23].

The potential for reducing SED was, however, substantial. At baseline, participants spent more than half of the 24-hour cycle sedentary. We identified several individualized facilitators and barriers [40] to design the intervention [21]. Still, the intervention did not change the behavior on average. The relationship between SED and cognitive functions is rather complicated. Cross-sectional evidence suggests SED is related to poor [5] and better cognitive functions [41]. A limitation in most studies is the reliance on self-reported measures of the behavior, which are known to have poor validity and reliability [8]. However, device-based measures (i.e., accelerometers) show no association between SED and cognitive functions among middle-aged adults [42] and among office workers, specifically [43]. Moreover, a recent systematic review of interventions aiming at reducing SED at work found no effect on cognitive functions [44].

To explore whether participants who changed behavior also changed cognitive functions, associations between changes in movement behavior and changes in cognitive functions were investigated. We found that neither changes in cardiorespiratory fitness nor any movement behavior were related to changes in any cognitive domain. The lack of association suggests that cognitive functions would not have been affected even if the intervention had successfully changed movement behaviors. A recent study indicated that the effect of these multi-component interventions was affected by the perceived work environment and was higher in individuals with higher executive functions [45]. This may partly explain the lack of association between changes in movement behavior and cognitive functions since it suggests that individuals who did succeed in changing their movement behaviors had higher executive functions at baseline and, therefore, had less possibility to improve cognitive functions further.

Both interventions and control groups improved cognitive performance from baseline to the 6-month follow-up on all cognitive domains. A retest effect may explain this increase. In a sensitivity analysis, we identified 38 persons who had already performed a similar neuropsychological test battery approximately 2 years before this data collection [43, 46] involving many of the same tests. Notably, these participants did not perform significantly differently from the rest of the sample at baseline or follow-up. In addition, changes from baseline to follow-up did not differ in this sub-sample. There are several reasons why retest effects might arise, such as reduced test anxiety and familiarization with the task [47]. The most pronounced retest effects occur from the first to the second testing session [48]. In the present study, 6 months separated baseline and follow-up testing, but we cannot exclude that retest effects might have affected the results. While we cannot exclude the possibility that the improvements in cognitive functions were related to an unknown confounding factor, our results suggest a learning effect in all three cognitive domains, even with the test being 6 months apart and alternative forms being used.

The association between physical activity and cognitive functions has been suggested to be influenced by several moderators, such as age, sex, and level of cardiorespiratory fitness [12], and researchers often adjust their models for these variables. However, the moderation effects of age, sex, and fitness level on the relationship between physical activity changes and cognition changes are rarely investigated [49]. The explorative moderation analyses revealed that age, sex, and cardiorespiratory fitness moderated the relationship between changes in some movement behavior ilr_1_ and some cognitive domains. However, these significant moderations should be interpreted carefully since the analyses were indeed explorative. Still, the results may motivate future investigations to assess whether the effects of changes in movement behaviors on cognitive functions vary by age, sex, and fitness level. Such examination may be helpful to identify target groups that would benefit the most from changing behavior.

In the present study, age moderated the relationship between changes in ilr_1_ Time in bed and changes in EF. This finding suggests that increasing Time in bed is related to more favorable changes in EF for those that were of higher age in our population. It is essential to acknowledge that we only assessed self-reported time in bed as a proxy for sleep-related behaviors in the present study. Thus, analysis of actual sleep duration or sleep efficiency as a measure of sleep quality may have clarified this moderation further.

Meta-analytic evidence shows that the efficacy of randomized controlled trials examining the effect of exercise modalities on cognitive function differs between sexes [11, 50]. We found that sex moderated the relationship between changes in ilr_1_ SED and changes in PS. Compared to females, increases in ilr_1_ SED were related to less improvement in PS among males.

We found that cardiorespiratory fitness moderated the relationship between changes in ilr_1_ MPA and changes in Global Cognition. Increases in MPA were favorably associated with improvements in Global Cognition for participants with higher compared to participants with lower baseline cardiorespiratory fitness. Meta-analyses consistently show that higher cardiorespiratory fitness is associated with a higher level of cognition in young adults and older adults [51, 52]. Notably, the relationship may not be linear as tests examining EF and EM only showed a significant relationship to 44 respective 43 mL·kg^− 1^·min^− 1^ of cardiorespiratory fitness [46].

In regards to the results from the exploratory moderation analysis, it is important to emphasise that any change-change association in the study population as a whole, or moderation thereof, is entirely disconnected from the randomized-controlled aspect of the study. In addition, the associated changes occur in the same time frame, which means that information regarding temporal precedence is also lacking. For these reasons, no causal inferences can be made in regards to this set of results.

### Methodological considerations

The neurocognitive battery consisted of several tests of many cognitive domains, making it possible to investigate latent cognitive constructs. However, the planned initial latent change scores did not work for us. Still, using cognitive composite scores of several tests made it possible to investigate domain-specific effects and Global Cognition. However, most studies investigating the impact of exercise on cognitive functions report only a few tests [53], making only test-specific investigations possible. Therefore, future studies examining the effect of changing movement behavior should continue to assess multiple cognitive domains, preferably with the modeling of latent constructs.

A limitation is the poor compliance of the included participants. Of the 263, only 139 participants remained and had valid accelerometer and neurocognitive data at follow-up. This imposes some concerns. First, the null finding of this study may stem from limited power to detect a difference between the groups. Second, the study participants remaining at follow-up may be a selected group.

## Conclusion

While structured exercise has been shown to convey broad improvements in cognitive function in inactive older adults, the effects of multi-component movement behavior change interventions on cognitive functions among sedentary office workers are largely unknown. Here we investigated the impact of two multi-component workplace interventions aiming to promote cognitive functions by reducing sedentary behavior or increasing physical activity among office workers. The lack of intervention effects on cognitive functions in this population of healthy office workers was in line with a previously reported lack of intervention effects on movement behaviors and cardiorespiratory fitness, which may be partly due to learning effects and selection bias (i.e., high baseline physical activity). Some associations between changes in cognition and changes in movement behavior were moderated by age, sex, and fitness level, which should be investigated more carefully to understand sub-groups who may benefit the most from changing specific movement behaviors. While these results suggest that it may not be worthwhile to provide this type of multifactorial support to already active office workers with the purpose of promoting cognitive function, it is still possible that such support would be effective in companies with less active employees. The study has demonstrated the challenges in achieving changes in movement behaviour in a workplace intervention, and thereby also difficulties in assessing the usefulness of such interventions for promoting cognitive functions. Future studies should carefully consider the need and potential for a successful intervention within the particular company and employee context, and direct systematic efforts into targeting workplaces with physically inactive workers.

## Data Availability

The datasets generated and/or analysed during the current study are not publicly available due to that the original approval by the regional ethics board and the informed consent from the participants do not include such direct free access, but are available from the corresponding author on reasonable request.

## Abbreviations

CBT: Cognitive behavioral therapy
CoDA: Compositional data analysis
EF: Executive function
EM: Episodic memory
ilr: Isometric log-ratios
LPA: Light physical activity
MI: Motivational interviewing
MPA: Moderate physical activity
MVPA: Moderate-to-vigorous physical activity
PS: Processing speed
SED: Sedentary behavior
VO_2max_: Maximal oxygen consumption
VPA: Vigorous physical activity
